# A comparative study of the efficacy of alginate lyases in the presence of metal ions elevated in the cystic fibrosis lung milieu

**DOI:** 10.1016/j.bbrep.2024.101821

**Published:** 2024-09-04

**Authors:** Neetu, T.N.C. Ramya

**Affiliations:** a CSIR- Institute of Microbial Technology, Sector 39-A, Chandigarh, 160036, India; b Academy of Scientific & Innovative Research (AcSIR), Ghaziabad, Uttar Pradesh, 201002, India

**Keywords:** Alginate lyases, Cystic fibrosis, Metal ions, Sputum, Biofilm, *Pseudomonas aeruginosa*

## Abstract

*Pseudomonas aeruginosa*, a common cause of morbidity in cystic fibrosis, chronically infects the patient's lungs by forming an alginate-containing biofilm. Alginate lyases are polysaccharide lyases that lyse alginate and are, therefore, potential biofilm-disruptive agents. However, cystic fibrosis sputum contains high levels of metals such as iron and zinc. The efficacy of alginate lyases under these conditions of elevated metal concentrations has not been categorically determined. Here, we have assessed the enzyme activity of two exolytic and five endolytic alginate lyases in the presence of metal ions (Fe^2+^, Zn^2+^, Mn^2+^, Mg^2+^, Ca^2+^, Ni^2+^, Cu^2+^) elevated in the cystic fibrosis lung milieu. Several of these alginate lyases exhibited increased activity in the presence of Ca^2+^, and the polysaccharide lyase family 7 members studied here exhibited decreased activity in the presence of Zn^2+^. The enzyme activity of the PL7 alginate lyases from *Cellulophaga algicola* (CaAly/CaFLDAly) and *Vibrio* sp. (VspAlyVI) was not affected in the presence of a mix of all the above-mentioned metal ions at the elevated concentrations found in the cystic fibrosis lung milieu. Specific alginate lyases might, therefore, retain the ability to degrade the alginate-containing *Pseudomonas* biofilm in the presence of metal ions elevated in the cystic fibrosis lung milieu.

## Highlights

• Modified Romling ASM was supplemented with metals to mimic cystic fibrosis sputum.
• Alginate lyases were assayed in the presence of metal ions elevated in CF sputum.
• CaAly, SA1-III, SA1-IV, VspAlylVI, PspAlgL, PspCY24AlyP1 and FspAlyFRB were studied.
• Ca^2+^ enhanced some alginate lyases and Zn^2+^ inhibited the PL7 members in this study.
• CaAly/CaFLDAly and VspAlyVI remained uninhibited by metal ions found in CF sputum.

## 1 Introduction

Cystic Fibrosis (CF) is a disease arising from mutations in the CFTR (cystic fibrosis transmembrane conductance regulator) gene and is characterized by impaired ion transport across epithelial cell membranes and consequent accumulation of mucus in airways. This compromised clearance mechanism creates an environment conducive to bacterial colonization, with *Pseudomonas aeruginosa* being a notable pathogen in CF lung [1]. The chronic presence of *P. aeruginosa* in CF lungs exacerbates inflammation, accelerates lung tissue damage, contributes to the decline in respiratory function, and results in loss of lung function. The abnormal anaerobic and low pH environment created by the dehydrated sputum drives the bacterial communities to adapt by growing as a mucoid form with enhanced biofilm formation [2,3] via the accumulation of a significant amount of alginate exopolysaccharide [4]. Alginate lyases are also referred to as alginases or alginate depolymerases and classified into polysaccharide lyase families (PL- 5, 6, 7, 14, 15, 17, 18, 31, 32, 34, 36, 38, 39, and 41) in the Carbohydrate Active Enzyme (CAZy) database [5]. They lyse the glycosidic bonds between alpha-l-guluronate/beta-d-mannuronate in alginate by a β-elimination mechanism, which results in varying sizes of oligosaccharides with 4-deoxy-alpha-L-*erythro*-hex-4-enuronosyl groups at the non-reducing ends [6]. Alginate lyases, in combination with antibiotics, have been studied for their ability to disrupt *P. aeruginosa* biofilms [7].

Measurements of airway secretions indicate increased levels of iron (Fe) and zinc (Zn) [8,9], besides modest increases in magnesium, calcium, and copper levels [10] in CF patients. Metal ions can interact with and play a significant role in protein structure stabilization and catalysis, and thereby enhance or inhibit enzyme activity; thus, the stability of alginate lyases in the presence of ions is an important characteristic to consider from the point of view of their value in disrupting *P. aeruginosa* biofilms in cystic fibrosis infections [11].

In this study, we aimed to assess the alginate lyase activity in ionic strength conditions of magnesium (Mg^2+^), manganese (Mn^2+^), calcium (Ca^2+^), iron (Fe^2+^), nickel (Ni^2+^), copper (Cu^2+^), and zinc (Zn^2+^) mimicking the cystic fibrosis lung milieu. These ions (other than Ni^2+^) have significant roles in inflammatory pathways in cystic fibrosis; Ni is a potential indicator of environmental contamination and lung fibrosis is one of the side effects of nickel contact [10,12]. We studied a set of previously characterized [13] seven different exolytic and endolytic alginate lyases from different polysaccharide lyase families, i.e., *Ca*Aly (alginate lyase domain) and *Ca*FLDAly (alginate lyase and F-type lectin domain) (PL7) from *Cellulophaga algicola*, *Psp*AlgL (PL5) from *Pseudomonas sp*. QD03, SA1-III (PL5) and SA1-IV (PL15) from *Sphingomonas sp.*, *Psp*CY24AlyPI (PL7) from *Pseudoalteromonas sp.* CY24, *Vsp*AlyVI (PL7) from *Vibrio sp.* QY101, and *Fsp*AlyFRB (PL15) lyase from *Falsirhodobacterium sp.*

## 2 Material and methods

### 2.1 Metal ion concentrations in cystic fibrosis

The highest reported concentrations of divalent ions observed in the cystic fibrosis lung were noted from the literature [10] (Table 1). For chloride salts of metal ions, magnesium chloride hexahydrate, calcium chloride dihydrate, copper chloride dihydrate, zinc chloride, nickel chloride, manganese chloride tetrahydrate, and ferrous chloride (all from Sigma) were used in this study at concentrations as mentioned in Table 1. For a mix of chloride/sulfate salts of metal ions, magnesium sulfate heptahydrate, manganese sulfate monohydrate, nickel sulfate hexahydrate, zinc sulfate heptahydrate, copper sulfate pentahydrate, ferrous sulfate heptahydrate, and calcium chloride dihydrate (all from Sigma) were used at the concentrations mentioned in Table 1.

**Table 1:** Table providing the metal concentrations reported in the cystic fibrosis sputum [10], metal ion concentrations (in mg/L) selected for this study, metal concentrations (in mg/kg) in ASM determined by Inductive Coupled Plasma- Mass Spectrometry in this study, and metal ion concentrations (in mM or μM) of the chloride/sulfate salts used in this study. BDL: Below Detection Limit with method detection limit of 0.1 mg/kg. In case of assays employing a mix of chloride and sulfate salts, Ca^2+^ alone was used in the chloride salt form.

Metal	Metal concentration in cystic fibrosis lung^10^ (mg/L)	Metal ion concentration selected for this study (mg/L)	Metal concentration in ASM (mg/kg)	Metal ion concentration for chloride/sulfate salts used in this study
Magnesium	19–44	50	8.36	2.05 mM (Mg^2+^)
Calcium	76–123	125	15.58	3.11 mM (Ca^2+^)
Copper	0.128–0.257	0.3	BDL	4.7 μM (Cu^2+^)
Nickel	0.01–0.06	0.06	BDL	1.02 μM (Ni^2+^)
Iron	0.398–1.292	1.3	1.66	24.10 μM (Fe^2+^)
Zinc	0.678–1.811	1.8	BDL	27.5 μM (Zn^2+^)
Manganese	0.004–0.017	0.02	BDL	0.36 μM (Mn^2+^)

### 2.2 Artificial Sputum Medium (ASM)

A modified version of the Artificial Sputum Medium developed by Romling was made [14,15]. Briefly, for 1 L ASM, 1.5 g mucin from porcine stomach (Sigma), 4 g low molecular weight DNA salmon sperm (Sigma), 5.9 mg Diethylene triamine penta-acetic acid (DTPA from Sigma), 5 g NaCl (Sigma), 2.2 g KCl (Sigma), and 1.81 g Tris base (Sigma), were dissolved in double distilled water and pH adjusted to 7.0. The mixture was then autoclaved and allowed to cool at room temperature. 5 mL egg yolk emulsion (Sigma) and 5 g mixture of all amino acids (casitone; pancreatic digest of casein; cat no 225930, BD Biosciences) were added into the mixture while maintaining pH and sterile conditions. ASM supplemented with ions was prepared by the addition of appropriate concentrations of all salts of metal ions other than Fe^2+^ in order to attain the final concentrations of metal ions, as mentioned in Table 1. Fe^2+^ salt was not added because the level of Fe^2+^ as measured by ICP-MS was higher than that selected for the study (Table 1). Chloride and sulfate salts were used at the concentrations mentioned in Table 1.

### 2.3 Inductively coupled plasma mass spectrometry (ICP-MS)

The metal ion concentrations in the ASM were determined by ICP-MS in accordance with the Association of Official Agricultural Chemists (AOAC) official method AOAC2015.01. Briefly, the sample was first digested with concentrated HNO_3_ and 30 % hydrogen peroxide by heating at 190 °C for a minimum of 10 min in microwave with the addition of Au to stabilize the Hg**.** The prepared sample was allowed to pump into the nebulizer so that the liquid sample would form an aerosol as it passed through the spray chamber, and was then transported into the high temperature plasma, where it was atomized and ionized, and detected by the detector on the basis of the *m*/*z* of the atoms.

### 2.4 Expression and purification of alginate lyases

All the proteins were expressed and purified as reported previously [13] and stored at −80 °C in Tris-buffered saline (20 mM Tris, 150 mM NaCl, pH 7.4) until their use in enzyme assays. The purified proteins were visualized by SDS-PAGE (**Supplementary Information**, Fig. S1).

### 2.5 Assessing alginate lyase activity

The activity of alginate lyases in the presence of each metal ion was assessed by setting up kinetic reactions to compare the alginate lyase activity as previously reported [16] in the presence and absence of each metal ion. The reaction was set up in a UV-compatible 96-well microtiter plate (Corning costar UV plate from Sigma) and contained 1 % sodium salt alginic acid (Sigma), 0.1 μM alginate lyase protein, and 1X metal ions (chloride salt) (as mentioned in Table 1) in Tris-buffered saline (TBS; 20 mM Tris, pH 7.4 and 150 mM NaCl). The initial velocity/rate of reaction catalyzed by the enzyme was assessed by measuring the absorbance at 235 nm, reflecting the concentration of the unsaturated product formed upon enzyme addition to the reaction mix. Absorbance was measured every minute at 235 nm wavelength at 37 °C in a Synergy H1 plate reader for 60 min, and converted into product concentration using the extinction coefficient, 6150 M^−1^ cm^−1^ [17]. The amount (nmoles) of product in the reaction volume of 100 μl was calculated from this and plotted against reaction time (in minutes) to yield the progress curve. The initial velocity of the enzyme reaction was then determined (as the slope, nmol product formed per minute) by fitting the initial linear region of the progress curve to a linear equation.

Similarly, the assay was set up with chloride salts of all seven metal ions simultaneously in TBS, and here the enzyme activity was measured at two different time points – 0 h and 6 h of enzyme incubation with the metal ions at 4 °C. The activity of the alginate lyases, SA1-III, *Fsp*AlyFRB, and *Psp*CY24AlyPI, was also determined after incubating them with chloride salts of metal ions in TBS for 6 h at 4 °C and subsequently dialyzing the proteins against TBS to remove ions. The enzyme activity of all alginate lyases was also determined following incubation for 6 h at 4 °C with chloride salts of all seven metal ions simultaneously in phosphate-buffered saline (PBS; 20 mM buffer comprising sodium dihydrogen phosphate and disodium hydrogen phosphate (both from Sigma), pH 7.4, and 150 mM NaCl) and HEPES-buffered saline (HBS; 20 mM HEPES (Sigma), pH 7.4, and 150 mM NaCl). The selection of 6 h (not a shorter or longer time period) was semi-arbitrary, considering that the time scale required for metal ions to bind to the protein molecules and affect their structure/function might range from minutes to hours. The respective buffer replaced alginate in the negative controls, and all the reactions were set up in triplicate with three biological replicates (three independent preparations of the alginate lyases). The paired *t*-test was applied to test for statistically significant differences (p-value <0.05) between the control and metal ion-added samples.

### 2.6 Thin layer chromatography

To check the activity of alginate lyase on ASM, 25 μL of 4X ASM (with or without supplementation of 4X appropriate concentrations of chloride/sulfate salts of metal ions required to achieve the sputum metal ion concentrations mentioned in Table 1) was diluted with 25 μL of 2 % alginate in TBS and added to 50 μL of 0.2 μM alginate lyases (to give a final concentration of 1X ASM and 1X metal ions) and incubated at 37 °C for 6 h. Then 5 μL of reaction mixture was spotted on silica gel 60 F 254, allowed to resolve in a solvent system comprising 1-butanol: acetic acid: water 3:2:2, then dried and stained with 1 mg/mL 1,3-Dihydroxynaphthalene (Sigma) (in 10 % sulfuric acid in 50 % ethanol) with heating over a hot plate for 10–15 min until bands became visible, as previously done [16]. For the initial experiment in Supplementary Information, Fig. S2, 25 μL of 2X ASM (without supplementation of metal ions) was diluted with 25 μL of 2 % alginate in TBS and added to 50 μL of 0.2 μM alginate lyases (to give a final concentration of 0.5X ASM and 0.5X metal ions) and incubated at 37 °C for different time points, then 5 μL of the reaction mixture was spotted on silica gel 60 F 254 and allowed to resolve in a solvent system comprising 1-butanol: acetic acid: water 3:2:2.

## 3 Results

### 3.1 Metal ion concentrations in ASM

Considering that ASM is used as a sputum substitute in studies, including those of cystic fibrosis, we first investigated whether the metal ion concentrations in the ASM formulation we used reflect the elevated metal concentrations observed in cystic fibrosis lung. ICP-MS analysis indicated that some metal concentrations in the ASM were significantly lower than those observed in the cystic fibrosis lung (Table 1). This suggested that the ASM would require supplementation with metals for studies intending to mimic the cystic fibrosis lung milieu.

### 3.2 Alginate lyase activity in ASM

**Fig. 1:**
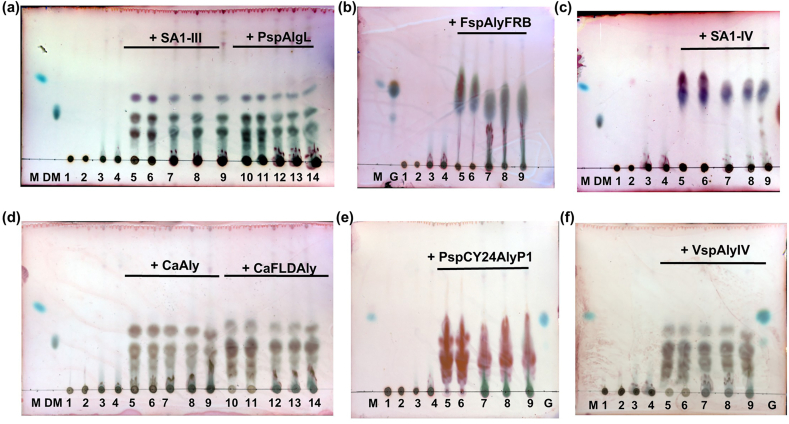
Activity of alginate lyases in ASM with or without supplementation of metal ions. (a–f) Thin Layer Chromatogram showing alginate lysis products of different lengths formed upon incubation of alginate with alginate lyases in buffer, ASM, or ASM supplemented with ions for 6 h at 37 °C. M: Mannuronate standard; DM: Di-mannuronate standard; G: Guluronate standard. Lane 1: Alginate in buffer; Lane 2: Alginate in buffer supplemented with chloride salts of metal ions; Lane 3: Alginate in ASM; Lane 4: Alginate in ASM supplemented with chloride salts of metal ions; Lanes 5 and 10: Alginate lyase plus alginate in buffer; Lanes 6 and 11: Alginate lyase plus alginate in buffer supplemented with chloride salts of metal ions; Lanes 7 and 12: Alginate lyase plus alginate in ASM; Lanes 8 and 13: Alginate lyase plus alginate in ASM supplemented with chloride salts of metal ions; Lanes 9 and 14: Alginate lyase plus alginate in ASM supplemented with chloride/sulfate salts of metal ions.

Next, we checked whether the alginate lyases were enzymatically active in ASM with or without supplementation with metal ions (to mimic the elevated metal concentrations in cystic fibrosis sputum) by visual inspection of the reaction products resolved by TLC. As observed in the Supplementary Information, Fig. S2, the TLC of the reaction mix of the alginate lyase *Ca*FLDAly showed increasing abundance of the alginate degradation products with time, with significant degradation observed in 6 h. Following incubation with alginate, the alginate lyases, *Fsp*AlyFRB and SA1-IV, showed a single spot as expected due to their exolytic nature, whereas the other alginate lyases showed multiple spots corresponding to alginate degradation products of different sizes (Fig. 1a-f). We found that all alginate lyase reactions in (1X) ASM as well as in (1X) ASM supplemented with chloride or sulfate salts (which more closely mimic the higher sulfate ion concentration in cystic fibrosis [18] of metal ions were grossly similar to control reactions (enzyme reactions in buffer lacking metal ions) (Fig. 1a-f). Hence, all the alginate lyases studied were similarly active in the presence and absence of (1X) ASM (even that supplemented with metal ions), and this suggested that the alginate lyases studied might be active even in the viscous, high ionic strength environment expected in cystic fibrosis sputum and lung.

### 3.3 Alginate lyase activity in the presence of single divalent metal ions

**Fig. 2:**
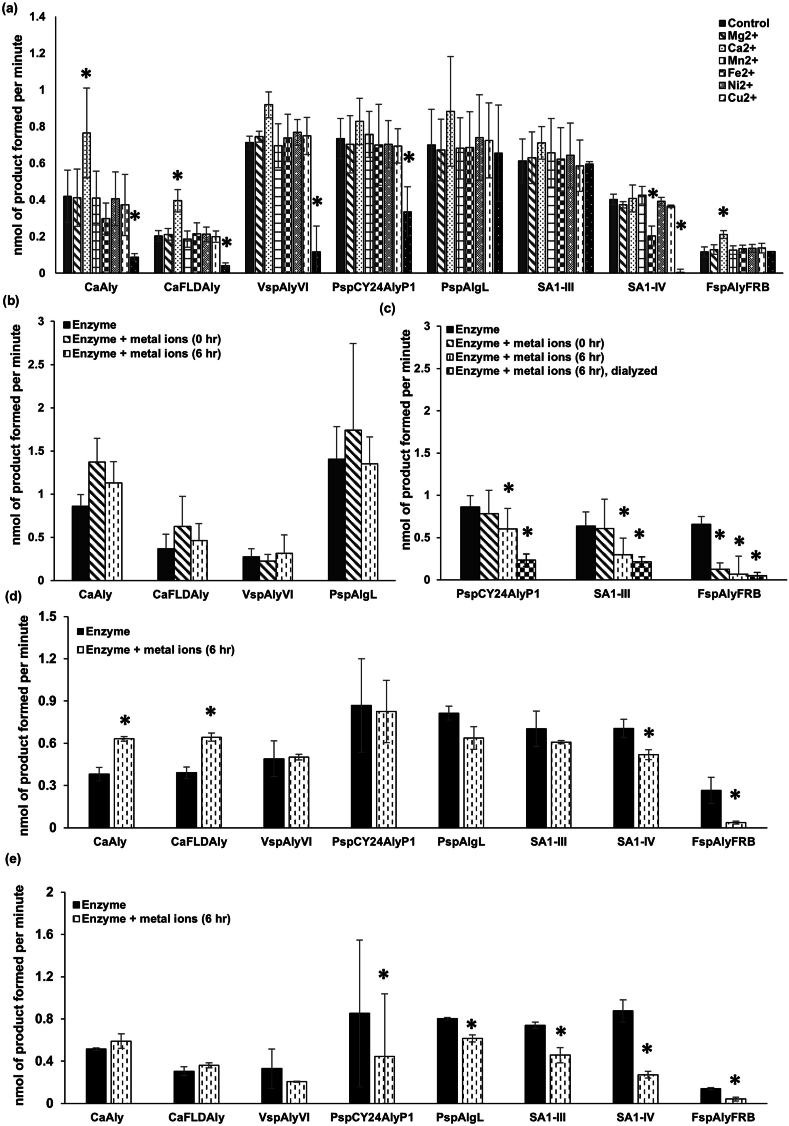
Alginate lyase activity in the presence of metal ions. (a) Alginate lyase activity in the absence or presence of chloride salts of individual divalent metal ions. The enzyme activity is represented by nmol product formed per minute in the reaction mix of volume 100 μL. Statistically significant changes (p-value < 0.05) are represented by asterisks. The experiment was conducted in triplicate with three biological replicates (three independent protein preparations). (b) Alginate lyase activity of *Ca*Aly, *Ca*FLDAly, *Vsp*AlyVI, *Psp*AlgL, and SA1-IV in the absence or presence of a mix of chloride salts of all divalent metal ions (with or without a 6-h incubation) in Tris-buffered saline. Buffer lacking metal ions was used for a control reaction. The enzyme activity is represented by nmol product formed per minute in the reaction mix of volume 100 μl. Statistically significant changes (p-value < 0.05) are represented by asterisks. The experiment was performed in triplicate with three biological replicates (three independent protein preparations). (c) Alginate lyase activity of *Psp*CY24AlyP1, SA1-III, and *Fsp*AlyFRB in the absence or presence of a mix of chloride salts of all divalent metal ions (with or without a 6-h incubation and/or dialysis to remove metal ions in case) in Tris-buffered saline. Buffer lacking metal ions was used for a control reaction. The enzyme activity is represented by nmol product formed per minute in the reaction mix of volume 100 μl. Statistically significant changes (p-value <0.05) are represented by asterisks. The alginate lyase activity in the absence and presence of all divalent metal ions with or without a 6-h incubation was measured twice in triplicate with three biological replicates (three independent protein preparations). (d) Alginate lyase activity in the absence or presence of a mix of chloride salts of all divalent metal ions (after a 6-h incubation) in phosphate-buffered saline. Buffer lacking metal ions was used for a control reaction. The enzyme activity is represented by nmol product formed per minute in the reaction mix of volume 100 μl. Statistically significant changes (p-value <0.05) are represented by asterisks. The experiment was performed in triplicate with three biological replicates (three independent protein preparations). (e) Alginate lyase activity in the absence or presence of a mix of chloride salts of all divalent metal ions (after a 6-h incubation) in HEPES-buffered saline. Buffer lacking metal ions was used for a control reaction. The enzyme activity is represented by nmol product formed per minute in the reaction mix of volume 100 μl. Statistically significant changes (p-value <0.05) are represented by asterisks. The experiment was performed in triplicate with three biological replicates (three independent protein preparations).

To further assess in detail the efficacy of alginate lyases in the cystic fibrosis lung milieu, we assayed the alginate lyases, *Ca*Aly, *Ca*FLDAly, SA1-III, SA1-IV, *Fsp*AlyFRB, *Psp*Cy24AlyP1, *Psp*AlgL, and *Vsp*AlyVI, in the presence of single divalent metal ions, magnesium (Mg^2+^), manganese (Mn^2+^), calcium (Ca^2+^), iron (Fe^2+^), nickel (Ni^2+^), copper (Cu^2+^), and zinc (Zn^2+^). Alginate lyases, *Ca*Aly, *Ca*FLDAly, *Vsp*AlyVI, and *Psp*Cy24AlyP1, belonging to class PL7 showed significant inhibition in the presence of Zn^2+^ (p-values of 0.037, 0.0063, 0.00042, and 0.039, respectively) (Fig. 2a). The PL7 alginate lyase domain-containing proteins from *C. algicola*, i.e., *Ca*Aly and *Ca*FLDAly, and the PL15 alginate lyase, *Fsp*AlyFRB, showed significantly increased activity in the presence of Ca^2+^ (p-values of 0.029, 0.0093 and 0.0051 respectively) (Fig. 2a). SA1-IV showed inhibition in the presence of Fe^2+^ and Zn^2+^ (p-values of 0.021 and 0.00065, respectively) (Fig. 2a). Class PL5 alginate lyases, SA1-III and *Psp*AlgL, and class PL15 alginate lyase, *Fsp*AlyFRB, did not exhibit inhibition in the presence of any of the metal ions selected (Fig. 2a).

### 3.4 Alginate lyase activity in the presence of a mix of all divalent metal ions

We next assessed the enzyme activity of the alginate lyases immediately upon and 6 h after the addition of a mix of chloride salts of all the seven divalent metal ions in TBS. The alginate lyases, *Ca*Aly, *Ca*FLDAly, *Psp*AlgL, *Vsp*AlyVI, and SA1-IV, were not inhibited by these treatments (Fig. 2b). *Fsp*AlyFRB showed a significant decrease in activity in both cases (p-values of 0.0079 and 0.0070, respectively), and SA1-III and *Psp*Cy24AlyP1 showed significant inhibition upon the 6-h incubation with ions (p-values of 6.1E-05 and 0.039, respectively) (Fig. 2c). The activity was not regained upon dialysis of the metal ion mix-incubated *Fsp*AlyFRB, SA1-III, and *Psp*CY24AlyP1 (p-values of 0.015, 0.00041, and 0.00017, respectively) against TBS, thus suggestive of tight binding of ions and/or irreversible changes in the overall structure or that of the active site (Fig. 2c). Considering that buffers might interact with metal ions and differentially affect enzyme activity, especially for metal-coordinated enzymes [19,20], we also determined the effect of a 6-h incubation with chloride salts of metal ions in PBS (Fig. 2d) and in HBS (Fig. 2e) on alginate lyase activity. *Ca*Aly and *Ca*FLDAly showed increased activity (with p-values of 0.015 and 0.022, respectively) whereas SA1-IV and *Fsp*AlyFRB showed significant decreases in activity (with p-values of 0.018 and 0.044, respectively) (Fig. 2d), and SA1-III, *Psp*CY24AlyP1, *Psp*AlgL, and *Vsp*AlyVI remained unaffected by a 6-h incubation with metal ions in PBS (Fig. 2c). Upon a 6-h incubation with metal ions in HBS, the alginate lyase activities of SA1-IV, *Psp*AlgL, *Fsp*AlyFRB, and *Psp*CY24AlyP1 were inhibited (with p-values of 0.0053, 0.019, 0.018, and 0.034, respectively); *Ca*Aly, *Ca*FLDAly, SA1-III, and *Vsp*AlyVI remained unaffected (Fig. 2d).

## 4 Discussion

Alginate lyases from various microorganisms, including *C*. *algicola* and *Pseudomonas TAG48*, have been demonstrated to have an anti-biofilm effect on *P. aeruginosa,* a common pathogen in cystic fibrosis patients [16,21]. SA1-III alginate lyase from *Sphingomonas* sp. was patented for its alginate lyase activity on cystic fibrosis mucus [22], and, alginate lyases, in combination with antibiotics, have shown promise in disrupting bacterial biofilms [7,11,23]. Considering their potential anti-biofilm role, it is relevant to explore if known alginate lyases can effectively function in the environment specific to the cystic fibrosis lung milieu that is characterized by viscous secretions and elevated metal concentrations.

ASM is a viscous sputum mimic that is used in studies to more closely reflect the physiological milieu, and different formulations of ASM have been reported with effects on *P. aeruginosa* secondary metabolites [24]. We used a modified version of the Artificial Sputum Medium developed by Romling [14,15] in this study and found that this ASM recipe requires supplementation with metal ions to actually mimic the high metal concentrations observed in cystic fibrosis sputum. Importantly, our study determined that seven distinct alginate lyases, *Ca*Aly/*Ca*FLDAly, SA1-III, SA1-IV, *Fsp*AlyFRB, *Psp*Cy24AlyP1, *Psp*AlgL, and *Vsp*AlyVI grossly retained the ability to degrade alginate in a viscous environment typified by ASM supplemented with chloride/sulfate salts of metal ions.

Following up on this positive finding, we also quantitatively assessed the effect of individual metal ions on these alginate lyases, and determined that whereas *Ca*Aly and *Ca*FLDAly (PL7) and *Fsp*AlyFRB (PL15) were enhanced by Ca^2+^, SA1-IV (PL15) and the PL7 alginate lyases, *Ca*Aly, *Ca*FLDAly, *Vsp*AlyVI, and *Psp*CY24AlyP1, were inhibited by Zn^2+^, and SA1-IV (PL15) was inhibited by Fe^2+^. Previous studies of several alginate lyases have demonstrated variable effects of various metal ions on enzyme activity, mostly with 1 mM metal ions, and indicate enhanced activity in the presence of Ca^2+^ in PL7 (*Ca*FLDAly [16], and alginate lyases from *Streptomyces* sp. M3 [25], *Vibrio* sp. W13 [26] and *Zobellia galactinovorans* [27]), and PL15 (SA1-IV [28]) enzymes. Variable activity has been demonstrated in the presence of Mn^2+^ - increased activity in SA1-IV (PL15) [28] and the PL7 alginate lyases from *Vibrio* sp. W13 [26] and *Streptomyces* sp. M3 [25] and decreased activity in the PL7 alginate lyase from *Microbulbifer* sp. SH-1 [29]. Variable activity has also been observed in the presence of Mg^2+^ - increased activity in SA1-IV (PL15) [28] and decreased activity in *Fsp*AlyFRB (PL15) (albeit with 30 mM Mg^2+^) [30] and the alginate lyase from *Vibrio* sp. W13 [26]. Fe^2+^ also elicits variable activity - increased in SA1-IV (PL15) [28] and decreased in *Ca*FLDAly (PL7) [16]). Reduced activity has been indicated in the presence of Zn^2+^ and Cu^2+^ in PL7, in *Ca*FLDAly [16] and the alginate lyases from *Streptomyces* sp. M3 [25] and *Vibrio* sp. W13 [26], and in *Ca*FLDAly [16] and the alginate lyase from *Vibrio* sp. W13 [26], respectively. Discrepancies probably reflect the different ion concentrations (as per actual cystic fibrosis lung milieu) employed in our study.

Interestingly, although *Ca*Aly, *Ca*FLDAy, and *Vsp*AlyVI were inhibited by Zn^2+^, they did not show any inhibition in the presence of the mix of all divalent metal ions (including Zn^2+^), and this is perhaps due to a compensating increase in enzyme activity brought about by other ions (such as Ca^2+^ in *Ca*Aly and *Ca*FLDAly). Considering the potential interaction of buffers with metal ions, and their effect on enzyme activity [19,20], we performed alginate lyase assays following a 6-h incubation in three buffer systems – Tris, phosphate, and HEPES, all at pH 7.4 and containing 150 mM NaCl, which is close to the physiological conditions observed in the cystic fibrosis lung environment [31,32]. Importantly, in our quantitative assays, the enzyme activity of the PL7 alginate lyases, *Ca*Aly, *Ca*FLDAly, and *Vsp*AlyVI, remained uninhibited in three different buffers (TBS, PBS, and HBS) containing the mix of chloride salts of the divalent metal ions, magnesium (Mg^2+^), manganese (Mn^2+^), calcium (Ca^2+^), iron (Fe^2+^), nickel (Ni^2+^), copper (Cu^2+^), and zinc (Zn^2+^), at the elevated concentrations found in the cystic fibrosis lung milieu.

To conclude, our study identifies alginate lyases that can efficaciously degrade alginate in the presence of metal ions elevated in the cystic fibrosis lung milieu. Efforts may be made in the future to further assess these alginate lyases for their potential as bio-therapeutic agents for treating *P*. *aeruginosa* infections in the airways of cystic fibrosis patients.

## Data availability

Data will be made available on request.

## CRediT authorship contribution statement

**Neetu:** Writing – review & editing, Writing – original draft, Visualization, Investigation. **T.N.C. Ramya:** Writing – review & editing, Supervision, Project administration, Funding acquisition, Conceptualization.

## Declaration of competing interest

The authors declare that they have no known competing financial interests or personal relationships that could have appeared to influence the work reported in this paper.
