# Supplementary material for: A comparative study of the efficacy of alginate lyases in the presence of metal ions elevated in the cystic fibrosis lung milieu
Source: Biochem Biophys Rep. 2024 Sep 4;40:101821. doi: 10.1016/j.bbrep.2024.101821 (PMC11404220; doi:10.1016/j.bbrep.2024.101821)
Supplement: Multimedia component 1 [file mmc1.docx]

**Supplementary Information**

**A comparative study of the efficacy of alginate lyases in the presence of metal ions elevated in the cystic fibrosis lung milieu**

Neetu^1,2^ and T.N.C. Ramya ^*,1,2^

^1^CSIR- Institute of Microbial Technology, Sector 39-A, Chandigarh 160036, INDIA

^2^ Academy of Scientific & Innovative Research (AcSIR), Ghaziabad, Uttar Pradesh 201002, India.

*Correspondence to be addressed to T.N.C. Ramya, CSIR- Institute of Microbial Technology, Sector 39-A, Chandigarh 160036, INDIA. Tel: 91-172-2880243; E-mail: ramya@imtech.res.in

Running Title: Alginate lyase activity in the presence of metal ions

Keywords: Alginate lyases, cystic fibrosis, metal ions, sputum, biofilm, *Pseudomonas aeruginosa*

**This Supplementary Data file contains two Supplementary Figures - S1 and S2.**

**Figure S1**


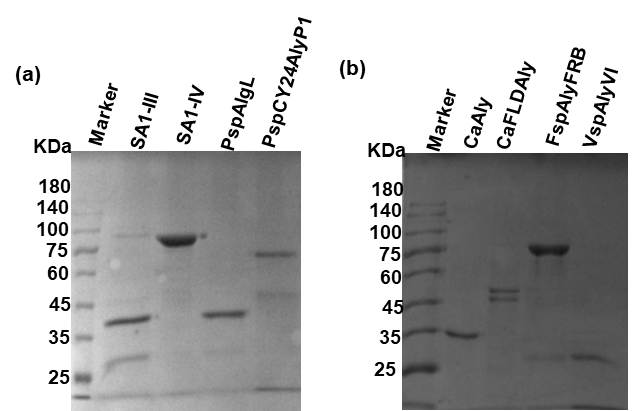


**Figure S1: SDS-PAGE of purified proteins.** (**a**) SA1-III, SA1-IV, PspAlgL, and PspCY24AlyP1. (**b**) CaAly, CaFLDAly, FspAlyFRB, and VspAlyVI.

**Figure S2**


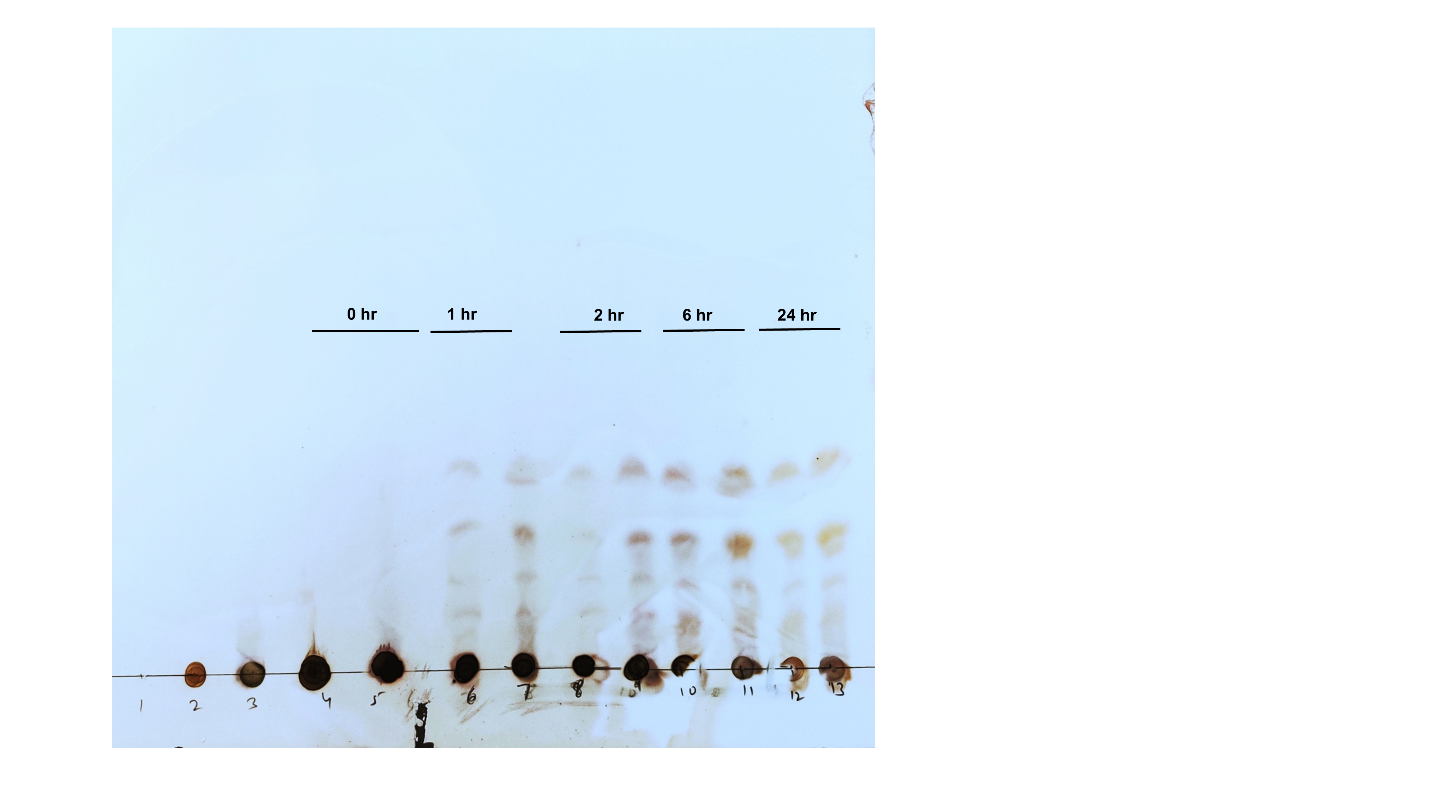


**Figure S2: Activity of CaFLDAly in alginate and 0.5X ASM.** Thin Layer Chromatogram showing alginate lysis products of different lengths formed upon incubation of alginate with CaFLDAly in buffer or in 0.5X ASM for different time points at 37 °C. Lane 1: CaFLDAly in buffer; Lane 2: Alginate in buffer; Lane 3: Alginate in 0.5X ASM; Lanes 4, 6, 8, 10, 12: CaFLDAly in alginate in buffer after 0 hours, 1 hour, 2 hours, 6 hours, and 24 hours, respectively; Lanes 5, 7, 9, 11, 13: CaFLDAly in alginate in 0.5X ASM after 0 hours, 1 hour, 2 hours, 6 hours, and 24 hours, respectively.
